# Effects of mindfulness-based stress reduction training on rumination in patients with breast cancer

**DOI:** 10.1186/s12905-022-02124-y

**Published:** 2022-12-28

**Authors:** Razieh Bagherzadeh, Rezvan Sohrabineghad, Taiebeh Gharibi, Farkhondeh Mehboodi, Hakimeh Vahedparast

**Affiliations:** 1 Department of Midwifery, Nursing and Midwifery Faculty, Bushehr University of Medical Sciences, Bushehr, Iran; 2 Department of Nursing, Nursing and Midwifery Faculty, Bushehr University of Medical Sciences, Bushehr, Iran

**Keywords:** Breast cancer, Mindfulness-based stress reduction, Rumination, Women

## Abstract

**Background:**

Breast cancer has been a serious public health dilemma for women worldwide, with not only physical and social impairments but also psychological stress responses such as rumination. Rumination is a constant preoccupation with thoughts. The present study aimed to investigate the effectiveness of mindfulness-based stress reduction training in lowering rumination among women diagnosed with breast cancer.

**Method:**

This randomized controlled trial with a pretest, posttest, control group, and one-month follow-up design included 46 female breast cancer survivors, recruited from the clinics and hematology wards of Bushehr, Iran. The inclusion criterion was that at least three months should have passed since the last chemotherapy/radiotherapy. The participants were randomly assigned to two experimental and control groups. The experimental group received eight sessions of mindfulness-based stress reduction training. A demographic information form and a rumination questionnaire were used for data collection, and the participants completed the questionnaire in the pretest, posttest, and follow-up stages. Chi-square, Mann–Whitney U, and repeated-measures ANOVA were used to analyze the data. *P* < 0.05 was considered statistically significant.

**Results:**

There was no significant difference in the rumination scores of the experimental group at three measurement stages. For the control group, the mean rumination scores on the posttest and follow-up were both significantly higher than on the pre-test (*P* < 0.001). The control group's mean follow-up rumination score was significantly higher than that of the post-test (*P* = 0.02). A comparison of the two groups adjusted for the baseline showed a significant difference between them in terms of the mean rumination score on the post-test (*P* = 0.01) and follow-up (*P* < 0.001).

**Conclusion:**

The experimental group was more successful in avoiding increased rumination than the control group, an ability that can be attributed to the effect of mindfulness training. The use of this method is recommended because it is non-invasive, non-pharmacological, free from complications, and can be easily performed by women. However, future studies should consider larger samples and long-term follow-ups.

## Background

Breast cancer (BC) is the most prevalent malignant type of cancer among women worldwide, with more than two million diagnoses in 2018 [1]. In the Eastern Mediterranean region which also includes Iran, cancer is a serious public health challenge with a heavy health and economic burden [2]. In Iran, BC is the most prevalent disease in women [3], while the most common cancers were breast, colorectal, and stomach in 2016 and are expected to remain the leading cancers nationally in 2025 [4]. Physical, mental, emotional, and social dysfunctions may ensue after the diagnosis of BC and the start of treatment, which are harmful to the general health of patients and disrupt their lives [5].

Additionally, negative metacognitive beliefs increase in women with BC [6]. Metacognitive beliefs are defined as one's beliefs about thinking and its processes [7]. There are two main metacognitive beliefs: positive and negative. Positive beliefs refer to the benefit of rumination (e.g., *If I am worried, I will be more prepared*), while negative beliefs refer to the uncontrollability and danger of rumination and its negative interpersonal and social consequences (e.g., *I have no control over my worry and rumination; feeling like this means I am losing my mind*). Rumination is a negative metacognitive state that negatively impacts mental health [6].

The response styles theory is the most robust and solid theory of rumination [8]. Based on this theory, when people are faced with unpleasant events, there are two main responses: distraction and rumination. A major characteristic of individuals who mostly use the rumination response style is assumed to be the fact that they ask a lot of questions in their minds about the possible causes of unpleasant events. They try hard to figure out the root(s) of that upsetting event, which means trying to solve the problem. But in doing so, not only do they fail to solve the problems, but also experience an increased level of anxiety and unusual worries [8, 9]. Patients with cancer were found to exhibit more negative metacognitive beliefs compared to healthy individuals [10]. Previous studies have shown that the prevalence of rumination is also high in women with BC [11–13].

Based on the estimation by the World Health Organization (WHO), the extent of psychological problems and concerns in patients with cancer is estimated to be 8 times higher than that in healthy people [14]; moreover, 80% of these patients feel concerned and frustrated and experience rumination in the first stages of their treatment [15]. Long-term rumination can harm health and well-being, which can lead to psychological distress and self-punishment behaviors [12]. According to the American Cancer Society, one of the most important factors in the survival of patients with BC is the assessment and management of long-term physical and mental health conditions related to the disease and its complications [16]. Compared to other complications of cancer or related treatments such as nausea, vomiting, hair loss, etc., the concept of rumination has received less attention from healthcare providers [17]. There is a concern about what treatment is more effective in reducing stress and rumination in cancer patients because these factors disrupt the quality of life and increase death anxiety [6]. To buffer these symptoms, complementary and alternative medicine is widely used by BC patients [18]. Based on studies, 32% of cancer patients report using mind–body interventions such as meditation to promote emotional and physical well-being and reduce psychological distress. Mindfulness-based stress reduction (MBSR) is one such intervention [18].

Mindfulness is a way of life that can be integrated into daily life through the practice of meditation. It helps individuals become familiar with the dual states of the mind and consciously use them as a cohesive mind [19]. Mindfulness means an awareness that emerges consciously by way of paying attention, in the present moment, non-judgmentally [20]. This attitude leads to awareness of negative thoughts and prevents rumination [6].

Various studies have investigated the effect of mindfulness on mental and physical variables in patients with BC and have reported different results. For instance, Campbell et al. [21] and Heydarian et al. [11] found that MBSR decreases rumination in women with cancer. However, Bisseling et al. [22] reported that MBSR had no effect on lowering rumination in BC patients. The results of a meta-analysis revealed that MBSR had no significant effect on anxiety, stress, pain, and the sleep quality [23].

A review of the literature revealed inconsistent results; in particular, there are few studies on this topic available in Iran as a country with unique cultural-religious characteristics. People's behavior and attitude towards illness and its treatment differ across cultures. As such, studies must be conducted in different cultures and societies [23]. Examining non-pharmacological strategies such as MBSR is essential for finding interventions to treat or prevent rumination. The ambiguities regarding relevant and efficient intervention methods to decrease patients’ rumination remain a challenge for healthcare providers, demonstrating the importance of research on non-pharmacological strategies such as MBSR for rumination. The present study, therefore, aimed to investigate the effectiveness of MBSR training on rumination in women with BC.

## Methods

### Design

This was a randomized controlled trial with an experimental and a control group, using the pre-test, post-test, and follow-up design.

### Patient population and sampling

The sample included all women with BC visiting the hematology wards of hospitals and hematology clinics in Bushehr, Iran, in 2018–2019. With the mean and standard deviation of rumination in the experimental (M = 45.10, SD = 37.83) and control (M = 7.53, SD = 5.97) groups based on Falsafi et al. [24], the alpha Type I error (α = 0.05), and 90% power, a sample size of 46 was obtained. After considering a 10% drop-out rate, a sample size of 52 (26 for each group) was determined. Convenience sampling was then performed, and 70 women with BC volunteered to participate in the study. Ten women did not meet the inclusion criteria, and eight of them refused to participate. Finally, 52 women were included. The participants were randomly assigned to the experimental and control groups by simple randomization using a random numbers table (26 participants per group).

### Inclusion and exclusion criteria

The inclusion criteria were consent and willingness to participate in the study, having BC for the first time, undergoing breast surgery or at least lobectomy, reading and writing literacy to fill out the questionnaires, having the physical ability to attend the meetings and doing the assignments, and at least three months having passed since the last chemotherapy/radiotherapy. The exclusion criteria were a history of mental illness, other serious malignancies, and insufficient Persian speaking or writing skills to participate in the study and fill out the questionnaires.

### Measures

The data collection instruments included a demographic information form and a rumination questionnaire. To measure rumination, the Rumination Questionnaire by Nolen-Hoeksema [8] was administered. It consists of 22 items, with each item scored on a four-point Likert scale ranging from 1 (never) to 4 (always). The total score of the questionnaire ranged from 22 to 88, and higher scores indicate greater degrees of rumination. The translation, cultural adaptation, and analysis of the psychometric properties of its Persian version were performed by Bagherinejad et al. [25]. The internal consistency of the questionnaire was confirmed with a Cronbach's alpha of 0.88. In Bagherinejad et al. study, the correlation between the scores of this questionnaire and those of depression and anxiety in a sample of Iranian students was 0.79 and 0.55, respectively [25].

### Data collection

The intervention was administered from 2018 to 2019. Eligible patients were asked to participate in the study. They received information about the trial and voluntary participation. They had the right to discontinue participation or withdraw their consent at any time. Then, patients who wanted to participate signed a written informed consent form before the start of the study. The patients willing to participate in the study were randomly divided into two groups. Then, they filled out the demographic information form and the rumination questionnaire.

The intervention was administered according to the Kabat-Zinn MBSR protocol [26]. This protocol allows attention to physical and environmental situations in the present moment and reduces automatic depressive processing. The primary mechanism of this treatment is self-control and self-attention because frequent focusing attention on a neutral stimulus such as breathing creates an appropriate attention environment [27]. As mindfulness training is better practiced with fewer individuals, the experimental group was divided into two groups (n = 13). The experimental group received eight 90-min sessions (one session per week) of MBSR, according to the Kabat-Zinn MBSR protocol. The contents presented in the training sessions are presented in Table 1 [28]. MBSR training was administered by a senior midwife who was an expert in MBSR and had experience working with women with cancer. She had a certificate of clinical competence and a master's degree in clinical psychology. The two groups were trained by the same tutor. To motivate the participants, the commuting and catering costs were covered by the research team. The intervention was provided in a quiet place designed for the mindfulness training, which was carpeted and equipped with chairs and visual learning aids.

**Table 1:** A review of the content of MBSR training sessions

First session	Introduction, introducing participants to each other, and starting conversations, the raisin meditation (eating raisins with the full involvement of the senses of smell, taste, sight and touch)
Second session	Body scan meditation, discuss the dealing with obstacles (such as restlessness and mind wandering), solutions (non-judgment and letting go of disturbing thoughts) and the difference between thoughts and feelings
Third session	Mindful seeing, smelling and listening (focusing of seeing, smelling and listening without any judgment or criticism)
Forth session	Sitting meditation with an emphasis on breathing*,* body sounds and thoughts (also called 4D meditation)
Fifth session	Sitting meditation, (on the subject of mindfulness about breathing, body, sounds, and thoughts), mindful movements, training and exercising the acceptance meditation and evaluating the negative automatic thoughts
Sixth session	Awareness of thoughts (thoughts are not facts,), allowing negative and positive thoughts to enter the mind and efficiently removing them from the mind
Seventh session	Taking care of yourself (what is the best way to take care of myself?), 4D meditation, and awareness about everything that comes to mind at the present moment
Eighth session	Sitting meditation, review all prior sessions and summarizing the program, discussion on how to incorporating mindfulness in daily life, congratulate yourself for reaching this point

During the 8 sessions, all the participants in the experimental group were requested to meditate for 10–15 min a day, 5–7 days a week. This time increased as the participants' experience increased every week. We used a notebook to track their assignments. The participants were asked to record their daily practice in a notebook. A WhatsApp group was created for the experimental group, the tutor, and the researchers to evaluate the completion of the assignment. The tutor was a facilitator who responded to questions and gave feedback if needed. The members were followed-up by giving reminders on WhatsApp.

No intervention was provided to the control group. To respect ethical considerations, at the end of the study, the participants of the control group who were willing to take part in the educational program received a pamphlet and a CD containing MBSR tutorials and were introduced to the MBSR training group.

A rumination questionnaire was filled out by both groups before the intervention, immediately after the intervention, and on the follow-up (one month after the intervention).

### Data analysis

**Fig. 1:**
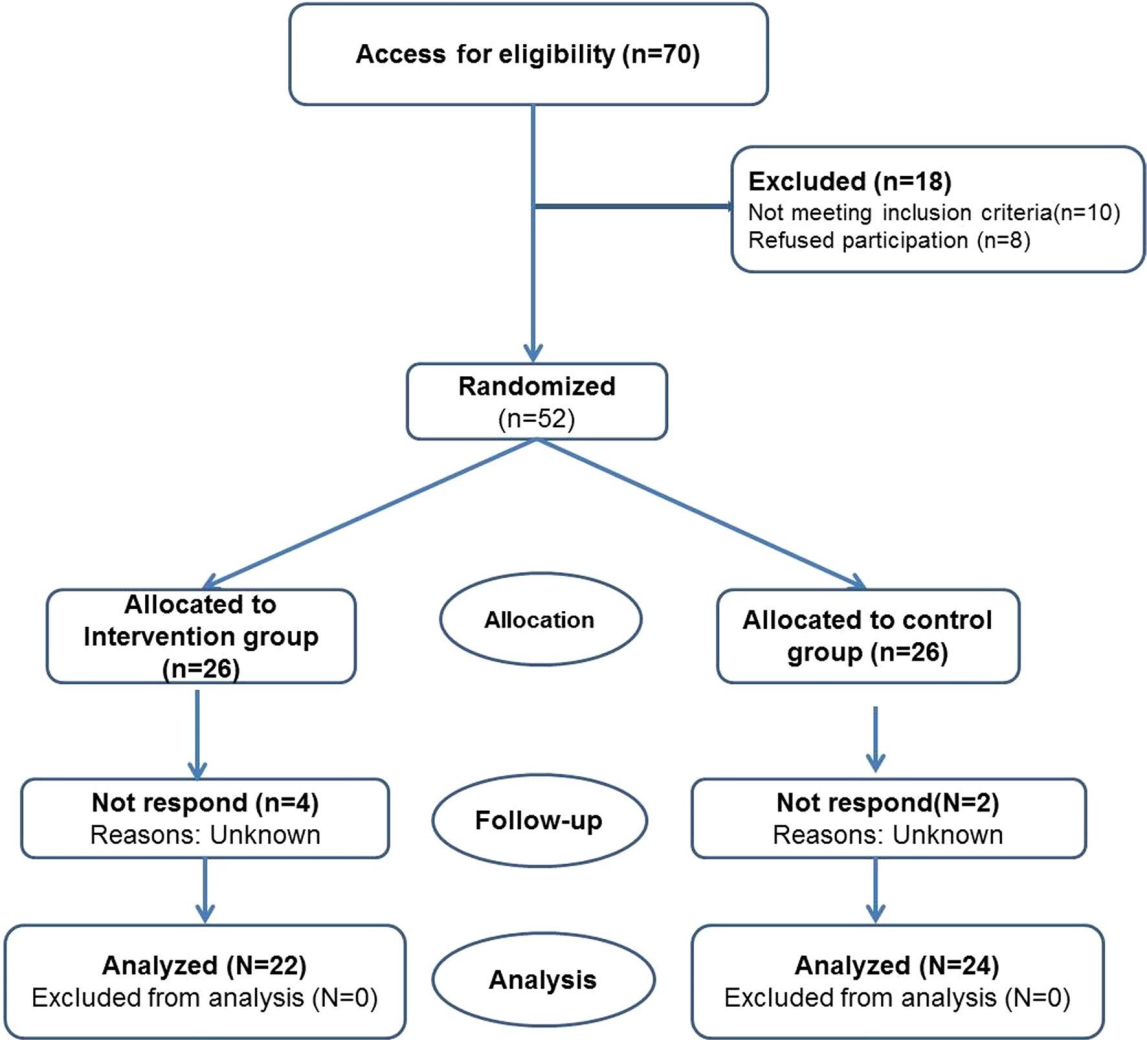
The consort diagram of the trial

At the outset of the study, the intention to treat analysis was taken into account. Out of 52 participants, two cases from the control group and four cases from the experimental group refused to continue the study, and the analysis was performed on 46 individuals (22 in the intervention and 24 in the control group) (Fig. 1). All the participants remained in their respective groups. All the participants in experimental groups (n = 22) participated in all the sessions. Therefore, the intention to treat analysis was similar to the per-protocol. The data were analyzed in SPSS 19. To evaluate the normality of distribution of the variables, the Shapiro–Wilk test was carried out. The variable of age did not follow a normal distribution, but rumination had a normal distribution (in two groups and at three evaluation times). In line with the research objectives, descriptive statistical indices (mean, standard deviation, percentage, frequency) and analytical tests, Chi-square and Mann–Whitney U, were used to compare demographic variables; moreover, repeated-measures analysis of variance (ANOVA) and analysis of covariance (ANCOVA) were performed for within- and between-groups comparisons. The assumptions related to the ANCOVA and repeated-measures ANOVA were also considered. The significance level was < 0.05. In the ANCOVA, the pretest score of rumination was considered as the covariance.

## Results

The mean age of the intervention and control groups was 44.50 ± 7.72 and 49 ± 7.48 years, respectively. There was no significant difference between the groups in terms of the mean age (*P* = 0.06, Z =  − 1.884) and the mean duration of the disease. Other demographic data were similar, and the groups were homogenous (Table 2).

**Table 2:** Comparison of demographic variables and the variables related to disease between intervention and control groups

	Intervention group frequency (%)	Control group frequency (%)	*P* value (Fisher or x^2^)
Education level			
Primary school	3 (13.6)	10 (41.7)	0.157 (5.17)*
Secondary school	3 (13.6)	3 (12.5)	
Diploma	5 (22.7)	5 (20.8)	
Academic	11 (50)	6 (25)	
Job			
Housewife	15 (68.2)	18 (75)	0.746 (0.263)
Employed	7 (13.8)	6 (25)	
Insurance			
Medical services	3 (13.6)	7 (29.2)	0.71 (5.17)*
Army	6 (27.3)	1 (4.2)	
Social security	13 (59.1)	16 (66.7)	
Breast surgery			
Bilateral mastectomy	10 (45.5)	15 (62.5)	0.303 (2.045)*
Unilateral mastectomy	1 (4.5)	0	
Lumpectomy	11 (50)	9 (37.5)	
Tamoxifen use			
None	5 (22.7)	12 (50)	0.69 (2.695)*
20 mg	5 (22.7)	3 (12.5)	
40 mg	12 (54.5)	9 (37.5)	

The statistic tests used is the chi-square or Fisher’s exact test

*Fisher exact test is done

The repeated-measures ANOVA showed that in the experimental group, the mean score of rumination did not significantly differ across three time points; but in the control group, the difference among three time points was statistically significant (Table 3). The post-hoc test demonstrated that the mean scores of rumination increased immediately and one month after the intervention compared to the baseline (*P* < 0.01). Moreover, the mean score of rumination in the control group significantly increased one month after the intervention compared to immediately after the intervention (*P* = 0.02) (Table 4).

**Table 3:** Comparison of scores of rumination before the intervention, immediately after and one month after the intervention in patients of control and intervention groups

Group	Before intervention	Immediately after intervention	1 month after intervention	Comparison of three times		
	Mean ± SD	Mean ± SD	Mean ± SD	Mean square	F	Sig
Intervention	43.18 ± 13.59	40.36 ± 10.28	40.82 ± 9.67	63.582	0.920	0.302
Control	42.96 ± 13.93	45.63 ± 12.54	47.29 ± 12.58	164.394	18.793	< 0.001

The statistical test used is repeated measures ANOVA

*P* < 0.05 is significant

**Table 4:** Comparison of both groups in terms of mean score of rumination immediately and one month after the intervention considering the impact of pre test

Group	Time	
	Immediately after the intervention	One month after the intervention
F value for the difference between both groups	7.348	15.179
Sig for the difference between both groups	0.01	< 0.001
F value for the impact of pre-test	84.424	125.474
Sig for the impact of pre-test	< 0.001	< 0.001

The statistical test used is analysis of covariance

*P* < 0.05 is significant

**Fig. 2:**
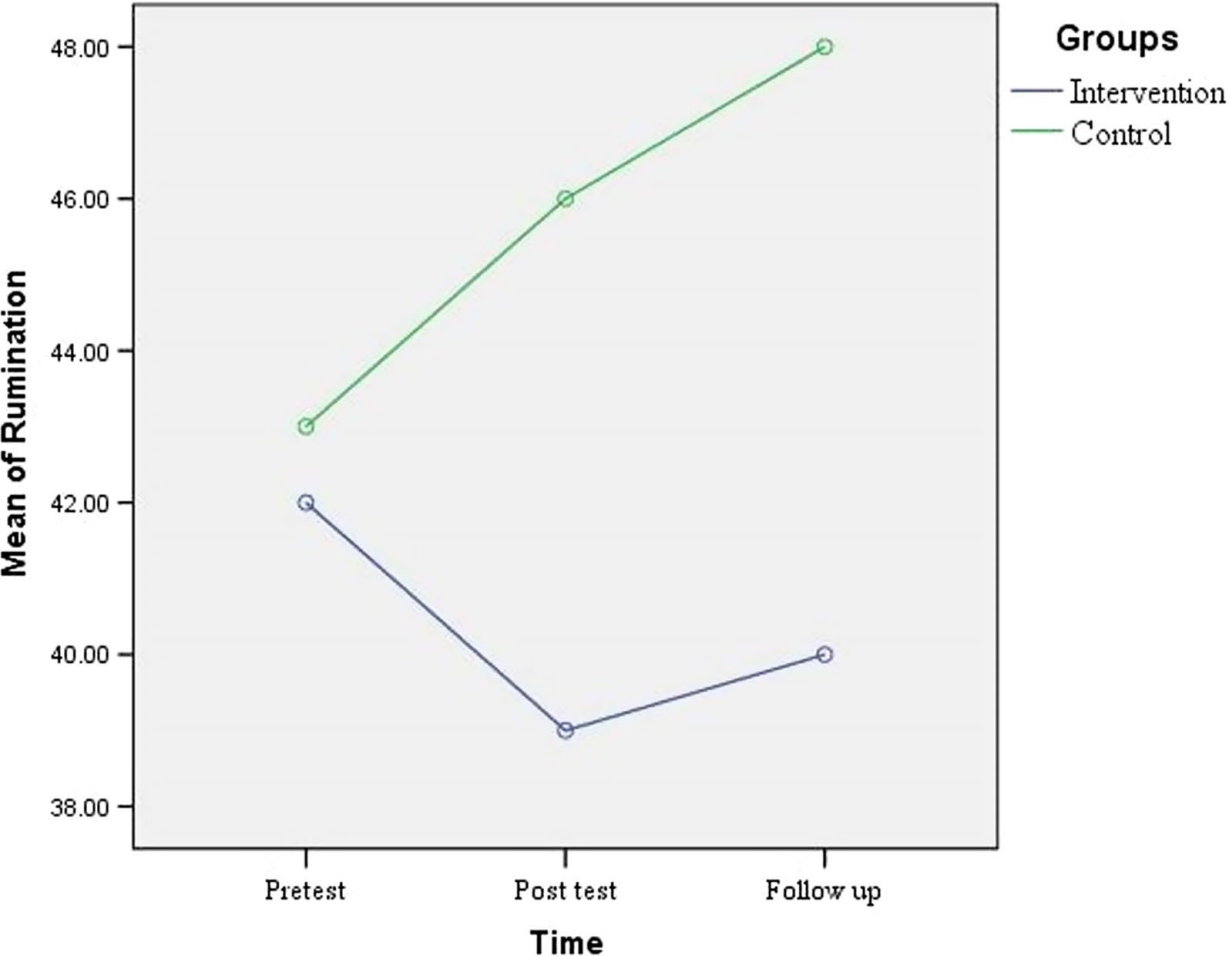
Mean rumination score over time in two groups

The results illustrate the ascending (significant) and descending (non-significant) trends of the rumination scores in the control and experimental groups, respectively (Fig. 2). Moreover, Fig. 2 shows that when the score is higher, rumination will be higher. Considering the effect of the pre-intervention, the comparisons suggested that there is a significant difference between the control and experimental groups regarding the rumination scores immediately and one month after the intervention.

## Discussion

This study aimed to examine the effectiveness of the MBSR program on the rumination of women suffering from BC. The results of the within-group comparison indicated that compared to pre-intervention, the mean score of rumination decreased in the experimental group after the intervention, but this difference was not statistically significant. In the control group, there was a significant difference among three time points, meaning that the scores of rumination had an ascending trend over time.

The between-group examination indicated a significant difference between the mean scores of ruminations across the two groups, which means that immediately and one month after the intervention, the mean score of rumination in the control group was more than that of the experimental group.

These results are in line with the findings of Bisseling et al. [22] in that MBSR did not reduce rumination. Considering the ascending trend of scores from pre-intervention to one month after the intervention in the control group and the lack of a difference in the experimental group, the result of the present study was inconsistent with that of Mohammadpour et al. [13] and Heydarian et al. [11], which showed no difference in different times in the control group and a decrease in the experimental group [11, 13]. The reason for this difference may be the primary scores of ruminations. The mean scores of rumination were higher in the study by Mohammadpour et al. This higher value could significantly decrease with interventions that are not long enough. On the other hand, rumination can be so high that it remains unchanged (stable) in the control group which is exposed to no intervention. In the study by Heydarian et al., although the intervention decreased the rumination, it failed to bring the rate to the average level or lower. The difference between the results of their study and the present study can be attributed to the use of different research instruments and the treatment status of the participants. The participants of their study were receiving treatment, or their cancer had metastasized, which can increase the rumination score. Moreover, Heydarian et al. used the Rumination Reflection Questionnaire, but the results were not reported separately by domains of the questionnaire (Self-Rumination & Self-Reflection); therefore, no comparison can be made with the current study.

The results of the present study, in which rumination was average, showed that if no intervention is offered to mitigate rumination in patients with BC, the level of rumination will rise. Although the intervention decreased rumination immediately after and one month after the intervention compared to pre-intervention, the amount of decrease was not significant. Furthermore, the results suggested that the MBSR intervention might have benefited from a longer exposure period. Although the rise in the mean score of rumination one month after the intervention is not significant compared to immediately after the intervention, rumination starts to increase if the intervention is stopped, which could be due to stopping mindfulness exercises after the intervention is over. This suggests the need for the intervention's continuity; so that the intervention becomes an integral part of the individual's daily behavior. A comparison of the effect of a mindfulness-based intervention on different levels of rumination can demonstrate how it affects groups with different levels of rumination; thus, the use of this intervention can be recommended to groups that benefit from it.

The between-groups analysis indicated that immediately and one month after the intervention, the mean score of rumination in the control group was greater than that of the experimental group. Although the decrease in the experimental group in the within-group analysis was not significant, the between-groups difference resulting from this decrease in the experimental group and an increase in the mean score of the control group showed that the experimental group was doing better than the control group in terms of rumination. These findings are consistent with some other studies [11, 13, 21, 24, 29, 30] which aimed to determine the effectiveness of mindfulness. Their results indicated the positive effect of mindfulness on decreasing rumination in patients with BC. Note that the difference between the two groups in the present study is due to an increase in rumination in the control group, not a decrease in rumination in the experimental group. Mindfulness decreases rumination and anxiety by teaching relaxation techniques, accepting the status quo without any judgment, and increasing awareness of the present moment [11]. Still, further research is needed to determine whether extending and continuing the intervention can decrease rumination. Moreover, using other therapies along with mindfulness and their comparison with this type of intervention can determine the best and the most effective intervention to address rumination resulting from BC and its complications.

The present study had some limitations. One of the most important limitations relates to the small sample size and, consequently, its limited statistical power capacity. Another limitation was the lack of long-term follow-ups due to the inaccessibility of all the participants during the period. Other studies should be conducted on larger samples, as well as with interventions and follow-ups over longer periods. Finally, as the stage of the disease was not considered in this study, further studies are advised to take it into account.

## Conclusion

The experimental group was more successful in coping with rumination than the control group, an ability that can be attributed to the effect of mindfulness training. Still, further research is warranted to determine whether extending and continuing the intervention can decrease rumination. In light of the increasing trend of BC in recent years and the higher predictability of this trend, other therapies can be used along with mindfulness, and their comparison with this type of intervention can help determine the best and most effective method to deal with rumination resulting from BC and its complications, thereby highlighting the need for further studies. Additionally, mindfulness training intervention with women suffering from BC can decrease the extent of rumination. Thus, it can be administered as a supportive treatment to improve the mental health of these patients throughout the treatment process. Since general healthcare providers are at the forefront of treatment and the ones patients normally visit, knowing about this issue would contribute to patients' recovery.

## Data Availability

The anonymized datasets used and/or analyzed during the current study are available from the corresponding author on reasonable request.
